# Snoring-generated fluid droplets as a potential mechanistic link between sleep-disordered breathing and pneumonia

**DOI:** 10.1186/s12931-024-02856-5

**Published:** 2024-05-29

**Authors:** Tayeb Kakeshpour, Kevin P. Fennelly, Adriaan Bax

**Affiliations:** 1 Laboratory of Chemical Physics, National Institute of Diabetes and Digestive and Kidney Diseases, National Institutes of Health, Bethesda, MD USA; 2 Pulmonary Branch, National Heart, Lung and Blood Institute, National Institutes of Health, Bethesda, MD USA

**Keywords:** Snoring, Respiratory aerosol, Self-infection, Pathogenesis, Pneumonia, COVID-19

## Abstract

The soft palate and back of the throat represent vulnerable early infection sites for SARS-CoV-2, influenza, streptococci, and many other pathogens. We demonstrate that snoring causes aerosolization of pharyngeal fluid that covers these surfaces, which previously has escaped detection because the inspired airstream carries the micron-sized droplets into the lung, inaccessible to traditional aerosol detectors. While many of these droplets will settle in the lower respiratory tract, a fraction of the respirable smallest droplets remains airborne and can be detected in exhaled breath. We distinguished these exhaled droplets from those generated by the underlying breathing activity by using a chemical tracer, thereby proving their existence. The direct transfer of pharyngeal fluids and their pathogens into the deep lung by snoring represents a plausible mechanistic link between the previously recognized association between sleep-disordered breathing and pneumonia incidence.

**Supplementary Information:**

The online version contains supplementary material available at 10.1186/s12931-024-02856-5.

## Introduction

Although snoring has long been linked to both chronic bronchitis in adults [1] and community acquired pneumonia in children [2], no mechanism for a causal relationship has yet emerged. Here, we present laboratory data supporting a mechanism whereby snoring generates pharyngeal fluid droplets that are carried, with their pathogens, deep into the lower respiratory tract (LRT) by the inspired airstream.

Radiotracer studies have shown that transfer of small amounts (≤ 100 µL) of oropharyngeal secretions into the lower respiratory tract (LRT) occurs at night during sleep in roughly half of healthy adults [3], a process generally referred to as microaspiration. Analogous import of oral microbiota into the lungs of healthy individuals has also been well documented [4]. Microaspiration is generally considered as the primary pathway for progression of upper airway infections to pneumonia, as documented for COVID-19 [5], but equally applicable to other bacterial and viral respiratory diseases [6]. Macroaspiration is commonly seen in obstructive sleep apnea (OSA) patients but involves the drawing of much larger quantities (> 0.5 mL) of fluid into the LRT [7], approximately doubling COVID-19 pneumonia risk [8]. 

While the word ‘microaspiration’ suggests a process analogous to macroaspiration, the sliding of tiny fluid droplets deep into the LRT, against the mucociliary clearance flow, defies the physics of fluids: Such micron-sized droplets would tightly adhere to the mucosal surface layer that covers the airways and not easily be moved by passing air. Moreover, deeper in the bronchial tree where the respiratory airflow invariably is laminar [9], surface-adhered particles cannot undergo net movements with the in- and outflow of passing air. By contrast, air can carry tiny fog-like droplets deep into the LRT. Only droplets smaller than about five micron will reach the lung parenchyma where their pathogen deposition can cause pneumonia [10]. Hence, we propose that both macro- and microaspiration include a pivotally important mode where droplets, generated by snoring, are carried by inspired air.

Snoring sounds result from soft tissue vibrations and the associated modulation of the airstream during inspiration, with anatomic details revealed by fiberoptic endoscopy [11]. Such video analysis showed periodic transient contacts between the back of the tongue and the soft palate, which flaps up and down driven by the inspired airstream. Fluid filaments are known to form between such transiently touching, wetted surfaces when they start to separate but are blown apart by the airstream, resulting in numerous tiny droplets. This droplet-generating mechanism was demonstrated to be highly productive for speech [12] but also is active during snoring. Because the inspired snoring airstream carries the particles first into the lung where they are inaccessible to traditional aerosol detectors, they could only be observed by generating an unnatural expiratory snoring sound [13]. Yet, following a regular inspiratory snoring sound, not all snoring-generated droplets “rain out” in the lung, and the fraction that remains airborne can be observed in subsequently exhaled breath. This exhaled fraction is analogous to cigarette smoke (particle size 0.1-1 micron) that is exhaled after first being drawn into the lung. Using chemical tracer technology, we here demonstrate that the exhaled snoring droplets are exclusively generated during inspiration when producing a snoring sound.

## Methods

**Fig. 1:**
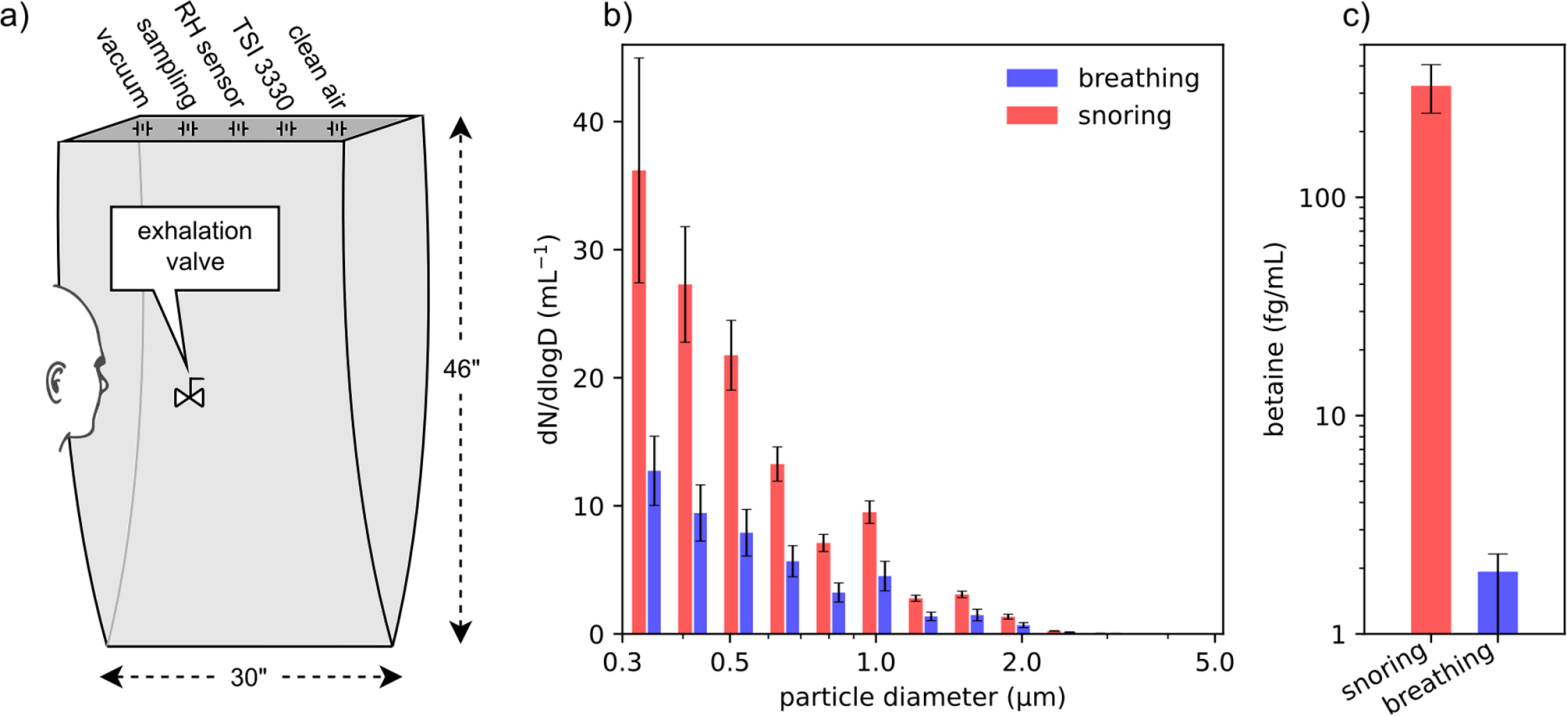
Measurement of aerosols emitted during wakeful voluntary snoring. (**A**) Experimental setup. The volunteer exhales through a valve into a polyethylene collection bag that is anti-static coated to prevent aerosol deposition. Ports at the top serve as inlets of ultra-clean dry air; a vacuum line to withdraw air for cleaning; a humidity sensor; a hose for sampling through a PTFE filter at a rate of 6 L/minute; and a connection to a TSI-3330 optical particle sizer that withdraws a ca. 14% fraction (1 L/minute) from the bag. (**B**) Comparison of the aerosol size spectrum of breathing and snoring maneuvers (66 ± 2 dBA at 50 cm), displaying the average count, *N*, as a function of diameter, *D*. (**C**) Comparison of betaine quantities sampled after breathing and snoring, presented on a log scale. Results are averages and standard deviations over five repeats, each consisting of 15 snores. Raw values and technical details are presented in Additional file 2

Measurements were carried out for a single healthy male volunteer (see Additional file 1) under an exemption from the NIH Institutional Review Board. To distinguish exhaled snoring aerosols from droplets generated in the lung by the underlying breathing activity, we periodically enriched the upper airway fluids with a concentrated solution of trimethyl-glycine (betaine; see Additional file 2). This food supplement, also naturally present at low levels in the body, served as a chemical tracer for aerosols generated by snoring in the pharynx. Expired air after each voluntary inspiratory snore was collected in a large volume bag (15 repeats) that had been pre-filled with 80L of ultra-clean dry air (Fig. 1a), thereby raising its humidity to *ca* 55%. Using a polytetrafluoroethylene (PTFE) filter, aerosols were collected from air extracted from the top of the bag, while monitoring its size distribution in parallel by an optical particle sizer (Model 3330, TSI Inc). By extracting air from the top of the bag, *ca* 23” above the breathing port, the bag also functioned as an effective saliva trap. After thoroughly cleaning the bag, the entire process was repeated while breathing at the same rate and volume. The mass of betaine captured in the PTFE filter was quantified by mass spectrometry. NMR measurement of the betaine concentration in throat fluid, collected by swabbing the back of the tongue immediately after each snoring and breathing maneuver, served as a reference for deriving the quantity of captured throat fluids. Experimental details are included in Additional file 2.

## Results

The size distribution of exhaled aerosols was found to be very similar for the snoring and breathing activities, but the number of particles observed after snoring inspiration was, on average, nearly three-fold higher (Fig. 1b). The presence of betaine in the snoring aerosols confirmed that these particles were generated in the pharynx. The absence of betaine in breath aerosol measurements (≤ 2 fg/mL) indicated that no above background betaine contamination of the snoring aerosols occurred from the underlying breathing activity (Fig. 1c).

The key question of where in the respiratory tract inhaled aerosols deposit has been extensively studied by use of radionuclides [14]. The site of deposition depends on respiration rate, respiration volume, and particle size. For aerosols in our observed size range, 0.3–2 μm, deposition is dominated by diffusion and gravitational sedimentation, and depends on the duration that they reside in any given area. Both modeling and experimental data showed that the vast majority (~ 90%) of such retained particles deposit in the alveolar ducts, alveolar sacs, and alveoli, but also that a substantial fraction (20–80%) of such particles escapes deposition and is subsequently exhaled [9, 14]. The latter fraction is observed in our measurements.

The similar particle size distributions observed for the breathing and snoring procedures (Fig. 1b) reflect filtering by the lung: breathing and snoring particles are both generated during inspiration [15], and only a relatively narrow spectrum of sizes, spanning from 0.01 to 2 μm, has a substantial probability to remain airborne during the breathing cycle [16] and therefore to be present in exhaled breath.

## Discussion

As observed in the activity of speaking, there can be substantial variations in aerosol quantities with loudness of snoring, with natural versus voluntary snoring, and between different persons. However, the physics underlying the generation of snoring sounds makes it impossible to snore without generating aerosols [12]. 

Snoring aerosols originate at the soft palate and the back of the throat, known to represent early infection sites for influenza [17] and SARS-CoV-2 [18], while also being vulnerable to other pathogens, including streptococcal Group A, measles, and respiratory syncytial virus (RSV). It therefore appears plausible that the snoring-induced transfer into the lung of pathogens, contained in the fluids covering these surfaces, will increase pneumonia risk.

Although the total fluid volume aerosolized by snoring is much smaller than for macroaspiration [7], the airborne route is known to be highly infectious [19]. In contrast to superspreader events, where only very few (ca. 0.1%) of the aerosols emitted by the index case are inhaled by any given participant [20], all droplets generated by snoring travel into the LRT where they potentially contribute to self-infection. Moreover, in contrast to partial virus inactivation from dehydration in the atmosphere [21], as applies for person-to-person transmission, virions in snoring droplets are not subject to such inactivation during their short path into the high humidity LRT, further increasing the risk of self-infecting the lung.

Even though our measurements suggest a physically realistic mechanism for direct airborne transport of pathogens into the lung parenchyma, more research is urgently needed to evaluate whether methods that prevent snoring in patients with an infection of the upper respiratory tract indeed reduce the incidence of progression to pneumonia.

### Electronic supplementary material

Below is the link to the electronic supplementary material.

Supplementary Material 1

Supplementary Material 2

## Data Availability

All data are contained in the Additional file 2 of the Electronic Supplementary Material.
