# Supplementary material for: Snoring-generated fluid droplets as a potential mechanistic link between sleep-disordered breathing and pneumonia
Source: Respir Res. 2024 May 29;25:224. doi: 10.1186/s12931-024-02856-5 (PMC11137920; doi:10.1186/s12931-024-02856-5)
Supplement: Supplementary file 1 — Supplementary Material 1 [file 12931_2024_2856_MOESM1_ESM.docx]

**Additional file 1: Volunteer health status**

The volunteer, male, age 67, was a never-smoker in good health with no history of asthma or obstructive sleep apnea, and a body mass index of 21. His forced vital capacity was 5.3 L; forced expiratory volume in 1 s (FEV1) 4.0 L; expiratory reserve volume 1.0 L.
