# Supplementary material for: Snoring-generated fluid droplets as a potential mechanistic link between sleep-disordered breathing and pneumonia
Source: Respir Res. 2024 May 29;25:224. doi: 10.1186/s12931-024-02856-5 (PMC11137920; doi:10.1186/s12931-024-02856-5)
Supplement: Supplementary file 2 — Supplementary Material 2 [file 12931_2024_2856_MOESM2_ESM.docx]

**Supplementary Materials and Methods**

***Breathing and snoring maneuver***

The volunteer was instructed to exhale to functional residual capacity (FRC), followed by slow (*ca* 3s) deep inhalation of *ca* 2.5 L of either snoring or mouth-breathing air, immediately followed (without closure of the glottis) by slow (3 s) expiration of 3 L of breath through the mouth into the collection bag. Each such maneuver was followed by a 10-second resting period before repeating. Immediately prior to the breathing or snoring maneuvers, the volunteer used a plastic pipette, to administer a concentrated betaine solution (2 mL, 1 M) to the back of the throat and tongue, targeting the areas that come into transient touching during snoring. Any excess solution was swallowed to cover regions inaccessible by pipetting. After each of three repeats, during the 10-s resting period the volunteer applied 1 mL of the betaine solution to the back of the tongue and swallowed. It was determined empirically that exhalation to functional residual capacity (FRC) followed by deep inhalation and exhalation to slightly below FRC was more robust and repeatable than controlling smaller volumes by digital spirometer feedback, which would involve travel through a 15-cm length flowtube. The latter also involved complications due to the change in temperature of the exhaled breath, and thereby further increase relative humidity, during travel through this tube, and therefore was not used. The timing of the maneuvers was controlled by a digital timer, and after a few practice runs the durations of inhalation and exhalation became reproducible to within 10%.

To prevent aerosolization of upper airway fluids during expiration, training of the volunteer to prevent transient closing/opening of the epiglottis during the lengthy breathing and snoring exercises was essential. Once trained, results proved highly reproducible.

The total volumes of observed breathing particles per liter of expired air (Table S1) were nearly five-fold higher than observed by Morawska et al.^1^ for much shallower, tidal breathing, but two-fold lower than observed by us for a deep exhalation maneuver.^2^

***Sampling reservoir***

Sample collections were carried out using a static-shielding polyethylene bag containing an aluminized polyester middle layer (30” width x 46” height, McMASTER-CARR, 4663T9). At the top of the plastic bag, five orifices were positioned for: (1) vacuum outlet for cleaning; (2) vacuum line for sampling; (3) rapid response relative humidity (RH) sensor (Honeywell, HIH-5030); (4) TSI-3330 optical particle sizer; and (5) ultra-clean anhydrous air inlet (**Scheme S1**). The top of the bag was reinforced with a horizontal aluminum sheet (24” x 6” x 3/8”) placed inside the bag, which had the above-mentioned ports precisely drilled through. The edges and corners of the aluminum sheet were rounded, and it was wrapped in static-shielding film to prevent damaging the bag. The bag was suspended inside a clean-air enclosure by hooks that were anchored in the aluminum sheet. A manually controlled rubber ball valve (inner diameter 1.1 cm; length 4.2 cm) at the front face of the bag served as a breathing port. The static shielding of the bag reduced loss of aerosols inside the bag over the 14-minute collection procedure from >90% to ≤10%, as monitored by the TSI-3330.


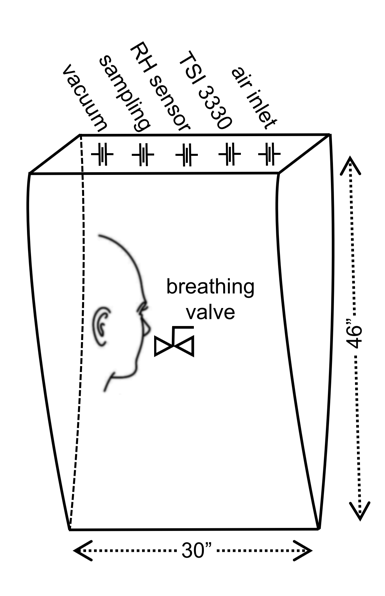


**Scheme S1.** Experimental setup for bioaerosol generation and collection

***Sample collection***

Prior to each sampling, a 3-micron PTFE filter (TISCH Scientific, SF18183) was freshly washed with ethanol (10 mL, 30% MQ water) and attached to the sampling port of the bag. The bag was then filled with 80 L of ultra-clean dry air, while the volunteer spent a minimum of one minute breathing inside the clean room before performing snoring or breathing maneuvers. Subsequently, a series of five snoring maneuvers were executed. Throughout the snoring actions, air was inhaled from the clean air enclosure and then exhaled into the collection bag. This entire procedure was repeated three times (resulting in a total of 15 snoring cycles), after which the humidity inside the bag reached 50-53%. This rise in humidity is equivalent to 27 L of exhaled air under the assumption that exhaled air is fully saturated at 35 °C. Upon the completion of the snoring maneuvers, an oral fluid sample was promptly collected from the back of the throat and tongue using a long cotton swab (MediChoice, WOD1004) for quantification of betaine in oral fluid by ^1^H NMR spectroscopy. Following a delay to allow precipitation of any spurious larger particles, air from the bag was drawn through the PTFE filter at a flow rate of 6 L/min for a duration of 14 minutes (totaling 84 L). During this sample collection, the particles inside the bag were also characterized using a TSI-3330 optical particle sizer, drawing air from the bag at a rate of 1 L/min (total of 14 L). For particle size distributions, see **Table S1**. For the control experiment, snoring actions were replaced by breathing at the same rate and volume.

***Quantification of betaine in oral fluid***

The mass of the oral fluid swab was determined gravimetrically from the difference between before and after swabbing. The contents of the swab then was mixed with D_2_O (0.7 mL). This solution was then transferred to a 5-mm NMR tube for analysis. A standard 1-D PRESAT experiment available from the Bruker library was used to suppress the residual water resonance. The concentrations of betaine in oral fluid samples are shown in **Table S2**.

***Sample processing and derivatization for liquid chromatography mass spectrometry (LC-MS)***

Betaine, a widely used food supplement, is a suitable chemical marker for oral fluids. Its nine chemically equivalent methyl protons yield a sharp singlet in the NMR spectrum, facilitating NMR detection but limited by the intrinsic low sensitivity of NMR relative to mass spectrometry (MS). Betaine’s quantification through MS, however, is hampered by its low pKa of 1.8, leading to a zwitterionic state with an overall neutral charge at pH levels compatible with most LC-MS instruments and columns suitable for its separation. To enhance its MS sensitivity, it was esterified to form a product with net positive charge using the reaction conditions outlined in **Scheme S2**.


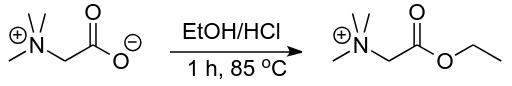


**Scheme S2.** Conversion of zwitterionic betaine to its positively charged ester derivate for enhanced quantification.

After sample collection, the trapped contents were eluted from the PTFE filters into total recovery LC-MS vials (Waters, 186000384C) by ethanol (400 μL, 30% MQ water) driven by a plastic syringe. Subsequently, the eluted solution was spiked with a betaine-*d_11_* solution (10 μL, 10 μM) followed by evaporation to dryness using a speedvac (ThermoSavant, DNA110). After that, the residue was resuspended in ethanolic HCl (50 μL, Sigma-Aldrich, 17934) and heated on a hot plate at 85 °C for 1 hour with the vial caps tightened (**Scheme S2**). After subsequent evaporation of all volatiles, the residue was resuspended in solvent A, comprised of 98% acetonitrile and 2% aqueous ammonium formate (200 mM, pH 3.0). A 5-μL portion of this solution was loaded into an analytical HILIC-Z column (2.1 x 50 mm, 2.7 μm, Agilent, 689775-924) that had been pre-equilibrated with solvent A at 15 °C. Elution was performed using an isocratic flow of the same solvent at a rate of 0.4 mL/min (Agilent, 1260 Infinity LC system). Quantification of the betaine ester (146.1 m/z) was achieved by comparing its peak area to that of the betaine-*d_11_* ester (157.2 m/z). Both chromatograms were obtained by the selective ion monitoring method using a single quadrupole mass spectrometer (Agilent, model 6130B) operating in positive ion mode with a fragmentor and capillary voltages of 120 and 1,500 volts, respectively. The electrospray nebulizer pressure was set to 35 psig and the drying gas flow and temperature were 12 L/min and 350 °C, respectively. The mass of betaine per total bag volume is listed in **Table S2** for each measurement.

**Table S1.** Number of particles per L of exhaled air detected by the TSI-3330 OPS.

| **bin**  **(micron)** |  | **breathing** | | | | |  | **snoring** | | | | |
| --- | --- | --- | --- | --- | --- | --- | --- | --- | --- | --- | --- | --- |
|  |  | **# 1** | **# 2** | **# 3** | **# 4** | **# 5** |  | **# 1** | **# 2** | **# 3** | **# 4** | **#5** |
| **0.30-0.37** |  | 756 | 1244 | 1180 | 1499 | 1419 |  | 3513 | 3704 | 2534 | 2691 | 4883 |
| **0.37-0.47** |  | 592 | 877 | 751 | 1124 | 1119 |  | 2788 | 2761 | 2149 | 2034 | 3172 |
| **0.47-0.58** |  | 484 | 811 | 622 | 916 | 929 |  | 2367 | 2231 | 1851 | 1683 | 2228 |
| **0.58-0.72** |  | 368 | 582 | 444 | 673 | 634 |  | 1446 | 1325 | 1205 | 1062 | 1277 |
| **0.72-0.90** |  | 199 | 337 | 247 | 375 | 374 |  | 744 | 734 | 669 | 568 | 653 |
| **0.90-1.12** |  | 265 | 471 | 340 | 521 | 553 |  | 1042 | 932 | 890 | 785 | 879 |
| **1.12-1.39** |  | 90 | 139 | 96 | 160 | 166 |  | 295 | 286 | 253 | 231 | 259 |
| **1.39-1.73** |  | 79 | 165 | 104 | 154 | 199 |  | 329 | 315 | 273 | 268 | 283 |
| **1.73-2.16** |  | 39 | 65 | 55 | 72 | 94 |  | 149 | 148 | 115 | 102 | 126 |
| **2.16-2.69** |  | 9 | 14 | 8 | 11 | 17 |  | 26 | 21 | 21 | 21 | 20 |
| **2.69-3.34** |  | 5 | 3 | 2 | 4 | 4 |  | 4 | 5 | 5 | 3 | 5 |
| **3.34-4.16** |  | 1 | 1 | 1 | 1 | 1 |  | 1 | 1 | 1 | 1 | 1 |
| **4.16-5.18** |  | 0 | 1 | 0 | 1 | 0 |  | 1 | 1 | 0 | 0 | 0 |
| **5.18-6.45** |  | 0 | 0 | 0 | 0 | 0 |  | 0 | 0 | 0 | 0 | 0 |
| **6.45-8.03** |  | 0 | 0 | 1 | 0 | 0 |  | 0 | 0 | 0 | 0 | 0 |
| **8.03-10.0** |  | 0 | 0 | 0 | 0 | 0 |  | 0 | 0 | 0 | 0 | 0 |

**Table S2.** Betaine contents of PTFE filters and oral fluid quantified by LC-MS and NMR, respectively.

| **experiment** | **betaine in aerosols (ng)^a^** | **betaine in oral fluid (mM)^b^** | **aerosolized oral fluid (pL/L)^c^** |
| --- | --- | --- | --- |
| **breathing 1** | 0.03 | 289 | 0.04^d^ |
| **breathing 2** | 0.06 | 291 | 0.08 ^d^ |
| **breathing 3** | 0.05 | 134 | 0.15 ^d^ |
| **breathing 4** | 0.05 | 211 | 0.10 ^d^ |
| **breathing 5** | 0.06 | 231 | 0.10 ^d^ |
| **snoring 1** | 9.77 | 150 | 26.7 |
| **snoring 2** | 11.12 | 84 | 53.4 |
| **snoring 3** | 5.46 | 143 | 15.4 |
| **snoring 4** | 6.71 | 114 | 23.7 |
| **snoring 5** | 10.17 | 101 | 40.6 |

**^a^** Total mass of betaine collected from the PTFE filter. For breathing measurements these are upper limits, determined by the baseline in the mass spectrum.

^b^ Concentration of betaine in oral fluid after 3 times 5 breathing and snoring maneuvers, with each set of 5 preceded by application, gargling, and swallowing of 2 mL of a 1 M betaine solution.

^c^ Estimated quantity of oral fluid captured by the PTFE filter in units of pL aerosolized oral fluid in 1 L of exhaled air.

^d^ Impurities present in the baseline of the mass spectrum may contribute to these values and reported quantities are upper limits for the true amount of aerosolized oral fluid during breathing.

**References**

1. Morawska L, Johnson GR, Ristovski ZD, et al. Size distribution and sites of origin of droplets expelled from the human respiratory tract during expiratory activities. *J Aerosol Sci* 2009;40(3):256-69. doi: 10.1016/j.jaerosci.2008.11.002

2. Shen Y, Courtney JM, Anfinrud P, et al. Hybrid measurement of respiratory aerosol reveals a dominant coarse fraction resulting from speech that remains airborne for minutes. *Proc Natl Acad Sci USA* 2022;119(26):e2203086119. doi: doi:10.1073/pnas.2203086119
